# Knowledge, Attitudes, and Behaviors around Dietary Fats among People with Type 2 Diabetes: A Systematic Review

**DOI:** 10.3390/nu16142185

**Published:** 2024-07-09

**Authors:** Justin Olive, Tommy Hon Ting Wong, Faye Chik, Sze-Yen Tan, Elena S. George

**Affiliations:** 1 Institute for Physical Activity and Nutrition, School of Exercise and Nutrition Sciences, Deakin University, Geelong, VIC 3220, Australia; justindanielolive@gmail.com (J.O.); fayechik@hotmail.com (F.C.); szeyen.tan@deakin.edu.au (S.-Y.T.); 2 School of Public Health, Li Ka Shing Faculty of Medicine, The University of Hong Kong, Pok Fu Lam, Hong Kong; tommyhtwong@gmail.com

**Keywords:** type 2 diabetes mellitus, knowledge, attitudes, behaviors, dietary fat, saturated fat

## Abstract

This systematic review assesses the knowledge, attitudes, and behaviors (KAB) surrounding dietary fat intake among people with type 2 diabetes mellitus (T2DM) and healthcare professionals. Following the Preferred Reporting Items for Systematic Reviews and Meta-Analyses guidelines, four databases were searched to identify studies published between 1995 and 2023 reporting people with T2DM or healthcare professionals that measured KAB towards dietary fat. This work was registered at PROSPERO (CRD42020140247). Twenty-four studies were included. Studies assessed knowledge of people with T2DM and reported poor nutrition knowledge regarding the health effect of fat consumption. Two opposing attitudes towards dietary fat was reported: (1) dietary fat should be limited, (2) promoted dietary fat intake through a low-carbohydrate diet. Participants reported behaviors of limiting fat intake, including trimming visible fat or choosing lower-fat alternatives. Total fat intake ranged between 10 and 66% of participants’ total energy intake, while saturated fat intake ranged between 10 and 17%. People with T2DM reported poor knowledge of dietary fats in particular, and they were frequently unable to identify high-fat food. Attitudes towards dietary fat were heterogenous, and regarding behaviors, saturated fat intake was higher than recommended. Future studies should assess the KAB of people with T2DM based on dietary fat subtypes.

## 1. Introduction

Type 2 diabetes mellitus (T2DM) remains a significant health issue around the globe [1], and dietary intervention is one of the first-line approaches for managing this condition [2]. In particular, the type of dietary fat is an important component of medical nutrition therapy and the management of T2DM [2]. Replacing saturated fat with mono- or polyunsaturated fatty acids (MUFAs and PUFAs, respectively) in the diet was found to be effective in the prevention and treatment of T2DM and its related complications [3,4], possibly through improving insulin sensitivity, reducing visceral fat deposits, and alleviating sub-clinical inflammation [5]. Furthermore, foods that are high in unsaturated fats, such as extra virgin olive oil, oily fish, nuts, seeds, and avocado, have been associated with benefits including glycemic management and improving metabolic and cardiovascular outcomes [6,7,8,9]. Unfortunately, dietary fat has long been stigmatized as an unhealthy component in the human diet, and the widespread belief that a low-fat diet is a healthy diet for people with diabetes is still commonly reported, albeit evidence has repeatedly demonstrated the benefits of incorporating unsaturated fats in a diet [10]. Furthermore, meta-analyses of randomized controlled trials have revealed that a diet restricting fat intake may not be beneficial [11] or might even be inferior in terms of metabolic outcomes when compared with diets that restrict carbohydrates [12]. Hence, the stigma related to total fat intake could be a major barrier to optimizing the dietary intake and increasing healthy unsaturated fat consumption in people with T2DM.

In individuals with T2DM, knowledge and attitudes are implicated in achieving desirable dietary behavioral changes, which, in turn, lead to treatment success and better health outcomes [2,13]. Specifically, individuals with chronic disease who identify barriers associated with their attitude towards healthy eating, such as “lack of willpower”, “time constraints”, or “time pressure” are more likely to have a poorer quality diet [14]. In addition to the known health benefits associated with healthy fats, dietary patterns rich in healthy fats have been reported to be more palatable and taste better [15]. Thus, there is an opportunity to provide dietary interventions and dietary guidelines that are more desirable and potentially sustainable for people with diabetes. To date there are limited studies assessing healthy fats and associated knowledge, attitudes, and behaviors (KABs) in people with diabetes. There is convincing evidence, including meta-analyses of correlational studies, to suggest that knowledge and the appeal and consequences of behaviors, namely attitudes, are reliable predictors of health behaviors [16]; however, there is no available review of these factors in the context of dietary fat in people with diabetes. Given the known health benefits of healthy dietary fats and diabetes, this poses a significant gap in the literature that may provide some direction for future study interventions and guide dietary recommendations. The aim of this systematic review was to synthesize the evidence surrounding KABs related to dietary fats in individuals with T2DM.

## 2. Material and Methods

### 2.1. Literature Search

This review was conducted in accordance to the PRISMA (Preferred Reporting Items for Systematic Reviews and Meta-analysis) guidelines (Table S1) and registered in PROSPERO (International Prospective Register for Systematic Reviews) under the registration code CRD42020140247. The research question was refined using the PICOS (Population, Intervention, Comparator, Outcome and Study Design) criteria (Table 1). A systematic search of papers published from January 1995 to October 2023 was conducted using four databases, MEDLINE, Embase, Cumulative Index to Nursing and Allied Health Literature (CINAHL), and PsycINFO. The search terms used are available in Table 2.

### 2.2. Eligibility and Selection

The eligibility criteria of this systematic review included the following: published between January 1995 and October 2023; available as full-text; written in English; assessed either or both of the following groups: healthcare professionals that work with adults (≥18 years old) with T2DM, or adults with prediabetes or T2DM; assessed knowledge, attitudes, or behaviors related to dietary fats and/or food groups that are regarded as rich in fat, e.g., meat, certain deep-sea fish, such as salmon and tuna, nuts, and seeds, as well as dietary patterns that were explicitly stated as low-fat or high-fat. Both qualitative and quantitative data were included. Articles were excluded if they were not conducted on humans, were review articles, contained the abstract only, or were not relevant to people with T2DM. Healthcare providers were included in the initial search but ultimately excluded from the review as there was only one paper yielded that met the eligibility criteria.

Title and abstract screening were performed independently by two researchers (JO, FC). Conflicting inclusions or exclusions between the two researchers were noted and individually resolved. The remaining studies were assessed for eligibility via a full-text screen, completed independently by two researchers (JO, FC). Conflicting results were resolved through consensus. Studies identified through hand-searching the reference lists of the included studies were also assessed and included through consensus.

### 2.3. Data Extraction and Synthesis

The following were extracted from each included study: name of the first author, year of publication, study design, participant characteristics (age, sex, and ethnicities), number of participants included, tools and methods for assessing KAB, KAB outcomes relevant to intake of the dietary fat or dietary patterns, and the country in which the study was conducted. Summary tables were created for knowledge, attitudes, and behaviors in separate tables presenting key study features and relevant findings.

### 2.4. Quality Assessment of Included Studies

Quality assessment was conducted for each included study using the Academy of Nutrition and Dietetics Evidence Analysis Library Quality Criteria Checklist [17]. This was completed independently by two researchers (JO, FC), and conflicts were resolved through consensus (Table 3).

## 3. Results

### 3.1. Study Selection

**Figure 1:**
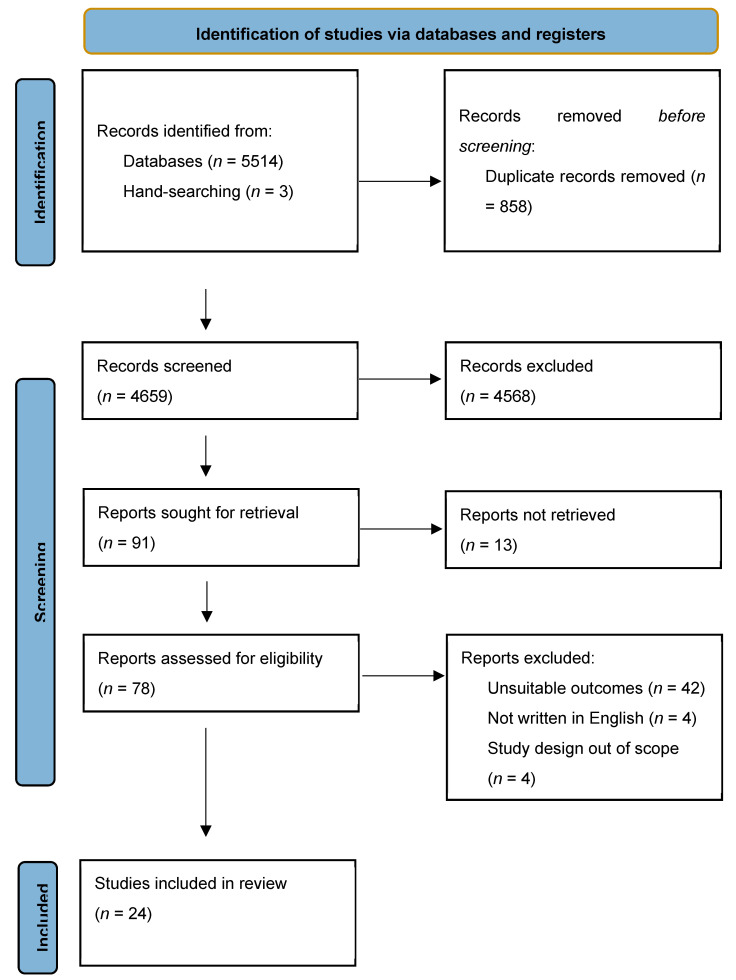
Study exclusion flowchart.

A total of 24 studies met the inclusion criteria and were included in this review (Figure 1). Based on the quality assessment, 13 studies received a positive rating, and 11 received a neutral rating.

### 3.2. Description of Included Studies

Study characteristics and the main findings are detailed in Table 4, Table 5 and Table 6. Twenty-three studies reported findings from individuals with T2DM (12–34), and one study involved dietitians (35). Four studies (13, 20, 22, 31) included comparisons pre- and post-intervention and the rest were cross-sectional in nature. Twelve studies were conducted in Europe, seven were conducted in the United States and Canada, and the rest in Asia and Africa.

### 3.3. Knowledge Relating to Dietary Fat

An assessment of fat-related knowledge of individuals with T2DM was conducted in four studies [18,19,40,41], and the findings are summarized in Table 4. Three studies required participants to identify foods that were rich in fat, and the results varied between studies. In a study conducted by Breen et al. [18], including 118 Irish adults with T2DM, more than 80% of participants were able to identify fried foods and pastries as high-fat foods. Conversely, a study conducted by Kessler et al. [41], including 190 adults in the United States (US) with either T1DM or T2DM, reported that fewer than half of the participants correctly identified foods that were high in fat. In line with this finding, Xue et al. [40] found that Chinese adults with T2DM performed the worst at identifying foods that were high in fat, compared to identifying foods that were high in carbohydrates or protein. All four studies also showed that participants had poor nutrition knowledge regarding the subtypes of dietary fat in food. Breen et al. [18] observed that fewer than half of the participants recognized that margarines and spreads have comparable energy contents with butter; only 30% of the participants were aware that not all fats and oils adversely affect cholesterol levels, and only 16% knew that the fat content in food does not directly influence blood glucose levels. In another study conducted by Devi et al. [19], including 340 Indian adults with T2DM, more than half of the participants were able to identify canola oil as “good oils”, but fewer than one-third of them recognized olive oil and safflower oil as beneficial and fewer than half of them identified butter, ghee, and coconut oil as “bad oils”. In the study conducted by Kessler et al. [41], fewer than half of the participants correctly answered the question regarding the “reduced fat” claim on food labels.

**Table 4:** Knowledge results.

First Author (Year of Publication)	Study Design	Population/Sample Involved	Country	Sample Size ^1^	Means/Instruments Used to Assess Knowledge	Main Findings
Breen (2015) [18]	Cross-sectional	Adults with T2DM Hospital; mean age 57.4 years old (SD 5.7), 64% male.	Ireland	118	Self-administered Audit of Diabetes Knowledge (ADKnowl) questionnaire. Total fat—1 question Fat subtype—2 questions	Over 80% of participants were aware that fried foods, pastry, and cakes are high in fat Only 44% knew that some margarines and spreads had comparable energy with butter 30% were aware that not all fats and oils adversely affect cholesterol levels Only 16% knew that the fat content of foods does not directly influence blood glucose levels
Devi (2021) [19]	Pre- and post-intervention comparison	Adults with T2DM residing in an East Delhi residential colony. Modal age group: 50–59 years old, 54.4% male.	India	340	Participants were interviewed using a pre-tested, semi-structured interview. Fat subtypes—1 question Identifying nuts and seeds—1 question	More than half of the participants (53–61%) were able to correctly identify canola oil as “good oils”, but few recognized olive oil (3–5%) and safflower oil (21–33%) Fewer than half of participants were able to correctly identify sources of bad oils, including butter, ghee, and coconut oil Patients’ performance improved after attending intervention programs
Xue (2020) [40]	Cross-sectional	Adults with T2DM recruited from six hospitals. Mean (SD) age: 59.1 (14.1) years old, 51.5% male.	China	334	Diabetes Dietary Knowledge Scale. Fat subtype—6 questions Nuts and seeds—2 questions	Participants performed the worst in identifying foods rich in healthy fats, compared with identifying foods that are rich in carbohydrates and proteins
Gebeyehu (2022) [25]	Cross-sectional	Adults with T2DM recruited from hospitals. Median age = 49.0 years old, 40% male.	Ethiopia	253	A face-to-face interview using a pre-tested, structured questionnaire and standard checklist. The questionnaire was adapted from previous studies and revised based on the objectives of the current study.	12.3% had “poor dietary knowledge” as a response for “Barrier to follow your dietary plan” 52.8% participants were found to have “poor” dietary knowledge; rated as <6 out of 11 questions correct

T1DM, type 1 diabetes mellitus. T2DM, type 2 diabetes mellitus. US, United States. ^1^ Only the number of participants fulfilling the inclusion criteria of this systematic review in each study was presented.

### 3.4. Attitudes Relating to Dietary Fat

Attitudes of people with T2DM towards dietary fat were assessed in five studies [21,31,33,38,39], while one study also assessed the attitudes of dietitians who had clinical experience with adults with T2DM [42]. Findings are summarized in Table 5, and the attitudes of people with T2DM towards dietary fat were highly heterogeneous. Participants in three studies perceived limiting fat intake to be coherent with a healthy diet or actively limiting fat intake in their diet [21,31,33]. In the remaining studies, participants who reported to be following a high-fat diet thought that following this dietary pattern was beneficial to their health [38,39]. They also reported difficulties in following a high-fat diet, including a lack of suitable food choices when eating out and meal preparation. In the study that assessed the attitudes of dietitians seeing people with T2DM [42], half of the dietitians reported that they prescribed a low-fat diet to their clients as they felt there were sufficient evidence to support this advice, while the rest expressed hesitancy to suggest a low-fat diet to their clients.

**Table 5:** Attitude results.

First Author (Year of Publication)	Study Design	Population/Sample Involved	Country	Sample Size ^1^	Means of Assessing Attitude	Main Findings
Ewers (2021) [21]	Cross-sectional	Adults with T2DM recruited from hospitals. Median (IQR) age of participants with T2DM: 66 (55–71) years old, 71% male.	Denmark	337	Structured face-to-face interview with open-ended questions. Participants were asked “What do you consider a healthy diet?” and then instructed to choose from a range of options.	16.6% of participants perceived a diet low in fat to be healthy 4.2% of participants perceived a diet including lean meat to be healthy 3.8% of participants perceived a diet high in fish to be healthy
Mphwanthe (2021) [31]	Cross-sectional	Adults with T2DM recruited from hospitals. Mean age (SD): 57.6 (8.3) years old, 46% male.	Malawi	39	Focus group discussions related to dietary habits for managing T2DM.	Participants commonly perceived avoiding oil/fat as a primary goal of their diet.
Parker (1995) [33]	Cross-sectional	Adults with T2DM recruited from a community intervention study. Mean age (SD): 47.4 (1.4) years old, 34% male, 87% white.	US	79	Structured interviews with 1 question related to limiting fat intake.	82% of the participants reported attempting to limit fat intake.
Webster (2019) [38]	Cross-sectional	Adults with T2DM following a diet program for >6 months. Mean (SD) age: 57 (10) years old, 50% male.	South Africa	28	Semi-structured, one-on-one interviews with a focus on low-carbohydrate high-fat diet	The following themes were identified from interviewing participants: Control of Eating: reduced hunger, reduced cravings for sweetness, reduced meal frequency, preference for “whole-foods”, increased perception of diet sustainability. Control of health: empowered and increased positivity, increased quality of life, increased perception of energy levels. Social eating: reduced enjoyment, negativity from public/peers.
Wong (2021) [39]	Cross-sectional	Adults with T1DM or T2DM who were on a ketogenic diet for ≥3 consecutive months. Mean (SD) age: 54.5 (10.1) years old, 43% male. T2DM *n* = 11.	Canada	14	Semi-structured interviews focusing on ketogenic diet	Motivators to start the diet: improve blood glucose control, reduce diabetes medications, and weight loss Participants reported positive effects on blood glucose control, reduction of diabetes medications, and weight loss Difficulty adjusting to KD was reported, due to the conventional belief that high-fat diets are unhealthy. This also prompted participants to develop new beliefs regarding a healthy diet Challenges of KD include limited food choices when eating out, difficulty in meal preparation, and keto flu Supports from healthcare providers were limited Information about KD lack credibility and was not evidence-based
Gebeyehu (2022) [25]	Cross-sectional	Adults with T2DM recruited from public hospitals. Median age = 49.0 years old, 40% male.	Ethiopia	253	A face-to-face interview using pre-tested, structured questionnaire and standard checklist. The questionnaire was adapted from previous studies and revised based on the objectives of the current study.	97% intended to cut down on fat/butter intake; “Did you cut down fat/butter intake?” -> “Yes”

IQR, interquartile range. T1DM, type 1 diabetes mellitus. T2DM, type 2 diabetes mellitus. US, United States. ^1^ Only the number of participants fulfilling the inclusion criteria of this systematic review in each study was presented.

### 3.5. Behaviors Relating to Fat Intake

A behavioral assessment relating to fat intake was conducted in 19 of the 24 studies that included individuals with T2DM [18,20,21,22,23,24,26,27,28,29,30,32,33,34,35,36,37,38,41], and the findings are summarized in Table 6. Main behaviors reported included the following: use of food labels for checking fat content, altering fat content in foods or one’s fat intake, and monitoring own fat consumption.

**Table 6:** Behavior results.

First Author (Year of Publication)	Study Design	Population/Sample Involved	Country	Sample Size ^1^	Means of Assessing Behavior	Main Findings
Breen (2015) [18]	Cross-sectional	Adults with T2DM recruited from hospital; mean age, 57.4 years old (SD 5.7), 64% male.	Ireland	118	Data of food label use were from the self-administered Audit of Diabetes Knowledge questionnaire. Dietary intake was recorded using a 4-day food diary, including at least one weekend day	49.2% of participants “often/sometimes” used food labels to check fat content Dietary intake in %E (mean (SD), low knowledge score vs. high knowledge score): ○ Total fat: 40.0 (6.9) vs. 39.2 (6.2) ○ MUFA: 13.8 (3.7) vs. 13.1 (2.6) ○ PUFA: 6.9 (2.2) vs. 7.0 (2.7) ○ SFA: 14.4 (3.0) vs. 13.8 (3.1) Dietary intake of total fat or fat subtypes did not differ with nutrition knowledge
Di Onofrio (2018) [20]	Pre- and post-intervention comparison	Adults with T2DM recruited from a nutritional motivational intervention study Intervention group: mean age (SD): 64 (6) years old, 68.1% male. Control group: mean age (SD): 65 (7) years old, 51.4% male.	Italy	279 (69 in the intervention arm and 210 in the control arm)	Questionnaire on dietary habits and behaviors	Percentage of participants that reported to regularly eat fish (baseline vs. follow-up): Intervention arm: 72.4 vs. 91.3 (*p* = 0.004) Control arm: 89.5 vs. 90.9 (*p* = 0.62) Fat intake of participants in intervention arm at baseline (mean) ^2^: Total lipids (g): 100.8 SFA (g): 34.2 PUFA (g): 9.4 MUFA (g): 49.0
Ewers (2021) [21]	Cross-sectional	Adults with T2DM recruited from an outpatient clinic. Median (IQR) age of participants with T2DM: 66 (55–71) years old, 71% male.	Denmark	337	Web-based semi-quantitative FFQ with 270 items	Intake of less healthy vs. healthy eaters (median and IQR): ○ Total fat (%E): 36.0 (32.4–40.6) vs. 36.9 (33.1–41.2) ○ SFA (%E): 13.4 (11.1–15.0) vs. 13.3 (11.3–15.3) ○ MUFA (%E): 13.6 (11.7–15.8) v 14.2 (12.2–16.2) ○ PUFA (%E): 6.3 (5.6–7.4) vs. 6.6 (5.5–7.5) ○ Fish (g/week): 173 (106–293) vs. 228 (127–336) Perceptions of dietary healthiness did not have a significant effect on intake of total fat, SFA, MUFA, or PUFA Self-rated “healthy eaters” had significantly higher (*p* = 0.006) intake of fish
Fitzgerald (2008) [22]	Cross-sectional	Adults with T2DM recruited aged between 35 to 60 years old. Mean age (SD): 48.9 (6.6) years old, 100% female.	US	100	FFQ with 18 items	Participants reported consuming meats less frequently than those without T2DM, but the difference was not statistically significant after adjusting for age and BMI
Fortes (2021) [23]	Cross-sectional	Adults with T2DM recruited aged 65 years old or recruited from an outpatient clinic, 52% aged between 65 and 74.9 years old, 53.7% male.	Italy	490	FFQ with 36 items	22.5% consumed nuts once or more than once per week, while the rest consumed less than weekly 94.5% reported exclusive use of olive oil 79.7% reported never using butter, 8.7% rarely or less than once per week, while the rest at least or more than once per week 28.6% consumed fish less than once per week, while the rest at least or more than once per week 60.3% consumed fish rich in *n*-3 fatty acids less than once per week, while the rest at least or more than once per week 15.5% consumed meat less than once per week, 44.5% 1–2 times/week, and the rest 3 times per week or more
Gauthier-Chelle (2004) [24]	Cross-sectional	Adults with T2DM, including males aged between 45 and 60 years old. Females aged between 45 and 60 years old; 61% male.	France	67	24 h recall	Dietary intake in males (mean (SD)): Total fat (g/d): 91 (4) Total fat (%E): 41.2 SFA (g/d): 38 (2) MUFA (g/d): 33 (1) PUFA (g/d): 13 (1) Dietary intake in females (mean (SD)): Total fat (g/d): 61 (4) Total fat (%E): 38.8 SFA (g/d): 25 (2) MUFA (g/d): 21 (2) PUFA (g/d): 9 (1)
Hendrychova (2013) [26]	Cross-sectional	Adults with T2DM recruited from outpatient clinic. Mean age (SD): 66.2 (10.1) years old, 55% male.	Czech Republic	200	Fat- and Fiber-related behavior Questionnaire	Women participants reported performing the following behaviors more often than men: modify meat to be low in fat avoid fat as flavoring replace high-fat meat with low-fat alternatives substitute specially manufactured low-fat foods replace high-fat foods with fruits and vegetables
Hendrychova (2015) [27]	Cross-sectional	Adults with T2DM recruited from various regions.	Czech Republic, US, Yemen	Czech Republic: 200; US: 207; Yemen: 200	Fat- and Fiber-related behavior Questionnaire	Fat-related behaviors were significantly different between countries. Participants from Czech Republic were most likely to replace high-fat meat with low-fat alternatives and substitute specially manufactured low-fat foods Participants from the United States were most likely to replace high-fat foods with fruits and vegetables Participants from Yemen were most likely to modify meat to be low in fat and avoid fat as flavoring
Ismael (2021) [28]	Pre- and post-intervention comparison	Adults with T2DM recruited from the MEDBIOME study. Mean age: 66 years old, male n = 6.	Portugal	9	Two non-consecutive 24 h recalls	Dietary intake (mean (SD)) at baseline: ○ Total fat (%E): 9.3 (4.6) ○ Total fat (g/day): 46.1 (36.3) ○ MUFA (g/day): 12.4 (9.2) ○ PUFA (g/day): 7.7 (6.2) ○ SFA (g/day): 17.1 (15.8) ○ Trans fatty acid (g/day): 0.9 (0.4) Percentage energy from fat intake increased from 9% at baseline to 20% after intervention Intakes of SFA, MUFA, and PUFA were similar before and after intervention Trans fat intake decreased from 0.9 g at baseline to 0.2 g after intervention
Lindstrom (2006) [29]	Pre- and post-intervention comparison	Adults with T2DM recruited into the Finnish Diabetes Prevention study. Mean age (SD): 55 (7) years old. Male n = 172. Participants in the control arm were given standard-care counselling, while those in the intervention arm were given individualized dietary counselling and physical activity sessions, and some also followed a very-low-calorie diet.	Finland	500	3-day food record at each visit	Dietary intake at baseline (mean (SD)) adjusting for intervention assignment and sex: ○ Total fat (%E): 37 (7) ○ SFA (%E): 17 (4) ○ MUFA (%E): 13 (3) ○ PUFA (%E): 6 (2) At baseline, percentage energy from total fat, SFA, MUFA, and PUFA did not significantly differ between those developed T2DM and those who did not Fat intake of participants with T2DM did not change significantly at follow-up
Melnik (2006) [30]	Cross-sectional	Adults with T2DM who were Puerto Rican living in New York City; 63.9% were 18–44 years old, 27.3% were 45–64 years old, 8.8% were 65 years old or older, 44.2% male.	US	606	Fat-related diet habits questionnaire	When compared with participants without T2D, those with T2D were more likely to modify meat to be low in fat, such as fat trimming or skin removal Scores for the following behaviors did not differ between participants with and without DM: avoid fat as a flavoring, avoid fried foods, substitute fat-modified food products, and replace high-fat foods with fruit and vegetables
Muñoz-Pareja (2012) [32]	Cross-sectional	Adults with T2DM recruited into the Nutrition and Cardiovascular Risk in Spain (ENRICA) study; 7.3% were 18–44 years old, 36.3% were 45–64 years old, and 56.4% were 65 years old or older, 59.1% male.	Spain	609	Computerized dietary history	Among all participants: 38.3% had less than 35% of total energy from total fat 37% had less than 10% energy from SFA intake, while 8.1% had less than 7% energy from SFA 80.1% had 10–20% energy from MUFA 96.1% had <10% energy from PUFA 88.3% used olive oil for cooking and 12% had more than 4 spoons of olive oil per day 90% had <1 serving of butter, margarine or cream per day 19% had nuts >3 times per week Dietary intake in %E (mean (SD)): Total fat: 36.7 (6.5) SFA: 11.2 (3.4) Trans fatty acid: 1.1 (0.6) MUFA: 16.1 (3.7) PUFA: 6.1 (1.9)
Parker (1995) [33]	Cross-sectional	Adults with T2DM recruited into the Heart Health Program in Pawtucket, Rhode Island, US. Age (SD): 47.4 (1.4) years old, 34% male, 87% white.	US	79	Semi-structured FFQ	Food intake in number of servings per week after adjusting for age, BMI, and energy intake (mean (SD)): ○ All red meats: 4.3 (0.4) All processed meats: 3.0 (0.5) Fish and poultry: 8.1 (0.5) Fats and oils: 17.3 (1.3) Participants with diabetes consumed more fish and poultry and fats and oils than those without diabetes, while intake of other food groups was similar Nutrient intake after adjusting for age, BMI, and energy intake (mean (95% CI)): ○ Total fat (g): 65.7 (62.5–69.1) ○ SFA (g): 23.3 (21.8–24.8) ○ MUFA (g): 23.9 (22.7–25.4) ○ PUFA (g): 11.7 (10.9–12.4) Participants with diabetes had higher PUFA, which was significantly higher in the comparison of fat subtypes (*p* = 0.01). Nutrient intake in percentage of total energy intake after adjusting for age and BMI (mean (95% CI)): ○ Total fat: 32.4 (30.9–33.9) ○ SFA: 11.7 (10.9–12.4) ○ Animal fat: 19.7 (18.4–21.1)
Quandt (2009) [34]	Cross-sectional	Adults with T2DM recruited into the Evaluating Long-term Diabetes Self-management among Elder Rural Adults (ELDERS) Study. Mean (SD) age: 74.1 (5.4) years old, 50.1% male. 31.4% were African American, 26.2% were American Indian, and the rest were White.	US	691	Fat- and Fiber-related behavior Questionnaire	The most reported behavior was avoiding fried foods The least reported behavior was for substituting fruits and vegetables for high-fat foods Women reported more common behaviors related to fat avoidance than men
Taylor (2014) [35]	Cross-sectional	Adults with T2DM recruited based on their attendance to prediabetes education classes between 2009 and 2011.	Canada	1228	Food Behavior Checklist	Male participants reported higher frequency of dietary behaviors consistent with greater saturated fat intake, including frying eggs in fat regularly, eating eggs with sausage, bacon, or ham regularly, and not regularly taking the skin off chicken.
Thewjitcharoen (2018) [36]	Cross-sectional	Adults diagnosed with T2DM attending outpatient clinics Mean (SD) age: 57.4 (10.9) years old, 47.4% male.	Thailand	304	3-day food record at Theptarin Hospital, and 7-day food record in Ramathibodi Hospital.	Dietary intake (mean (SD)): ○ Total fat (g/day): 49 (20) ○ Total fat (%E): 31 (7) ○ SFA (%E): 9.8 (5.6) Only 32.7% of all participants met recommendations for saturated fat (<10% of total energy) Fat intake did not differ between those with good and poor glycemic control 34% of participants “often” or “always” attempted to reduce fat intake, while 27% of participants suggested they “never” or “seldom” did so.
Vasconcelos (2021) [37]	Pre- and post-intervention analysis	Adults with T2DM recruited aged between 50 and 80 years old were selected by medical doctors. Mean age (SD): 65.4 (5.9) years old, male n = 19.	Portugal	33	3-day food diary (2 weekdays and 1 weekend day)	In the control group, percentage of energy from PUFA decreased at follow-up, while those for total fat, saturated fat, and MUFA were not significantly different In the experimental group, percentage of energy from total fat and PUFA increased at follow-up, while those of saturated fat and MUFA was not significantly different
Webster (2019) [38]	Cross-sectional	Adults with T2DM recruited following a diet for >6 months Mean (SD) age: 57 (10) years old, 50% male.	South Africa	28	Types of food eaten were assessed by FFQ. Nutrient composition and energy intake were quantified using a 1-day food recall and a 3-day food record.	Dietary intake (median (IQR)): ○ Total fat (%E): 66 (62–69) ○ Total fat (g/day): 121 (99–176) ○ SFA (g/day): 49 (40–73) ○ MUFA (g/day): 45 (37–60) ○ PUFA (g/day): 13 (9–21) Dairy food was one of the most commonly consumed food groups Participants reported eating fatty cuts of meat and full-fat dairy, while lean meats and low-fat dairy were rarely reported
Gebeyehu (2022) [25]	Cross-sectional	Adults with T2DM recruited from local hospitals. Median age = 49.0 years old, 40% male.	Ethiopia	253	A face-to-face interview using pre-tested, structured questionnaire and standard checklist. The questionnaire was adapted from previous studies and revised based on the objectives of the current study.	“Type of oil used for cooking food” Saturated fatty acid: 42.2% Unsaturated fatty acid: 57.8%

%E, percentage of total energy intake. BMI, body mass index. FFQ, food frequency questionnaire. IQR, interquartile range. MUFA, monounsaturated fatty acid. PUFA, polyunsaturated fatty acid. SFA, saturated fatty acid. T2DM, type 2 diabetes mellitus. US, United States. ^1^ Only the number of participants fulfilling the inclusion criteria of this systematic review in each study was presented. ^2^ Data of control group were not presented.

Two studies investigated food-label use in adults with T2DM, one in Ireland [18] and one in the United States (US) [41]. Both studies observed that more than half of the participants “often/sometimes” used food labels to check fat content of packaged food. Six studies recorded participants’ behaviors to alter either visible fat with food or their intake of dietary fat [26,27,30,34,35,41]. One study performed on Puerto Rican adults (n = 606) residing in New York City, US, found that those with T2DM were more likely to remove the skin or trim the fat off the meat than those without. Kessler et al. [41] surveyed the dietary behavior of a group of adults (n = 190) with either T1DM or T2DM and found that 62% of the participants reported limiting their total fat intake. Quandt et al. [34] reported that the most common strategy to reduce fat intake was avoiding fried foods, while the least commonly reported behavior was substituting fruits and vegetables for high fat foods. Hendrychova et al. [27] compared behaviors of people with T2DM among US, Czech Republic, and Yemen. They noted that participants from both Czech Republic and US were most likely to replace high-fat meat with low-fat alternatives, while participants from Yemen reported trimming off visible fat and avoiding fat as flavoring to be the common practices. However, in another study conducted by Thewjitcharoen et al. [36] involving 304 Thai adults with T2DM, only 34% of the participants with T2DM reported often or always attempting to reduce fat intake. In terms of behaviors related to fat intake, there were some differences between males and females, where three studies consistently observed that females were more likely to actively limit their fat intake than males [26,34,35].

Twelve studies assessed fat intake of people with T2DM [18,20,21,22,23,24,28,29,32,33,36,38]. The intake of total fat ranged from 10 to 66% of total energy, intake of saturated fat ranged from 10 to 17%, intake of monounsaturated fat from 13 to 24%, and intake of polyunsaturated fat from 6 to 12%. Five studies also assessed the intake of various food groups in participants with T2DM [21,22,23,32,33], and the findings varied between studies. Fish consumption was often recorded among participants with T2DM. Ewers et al. [21] observed in a group of Dane adults with T2DM that those who self-rated as “healthy eaters” consumed more fish than “unhealthy eaters”. Fortes et al. [23] observed in a group of Italian adults that 70% of participants reported consuming fish at least once a week. Similarly for meat, Fitzgerald et al. [22] observed that participants with T2DM consumed meat less frequently than those without T2DM. Nuts were reportedly not consumed regularly by people with T2DM: Fortes et al. [23] observed that only 22.5% of their study participants consumed nuts once or more than once per week. Munoz-Pareja et al. [32] observed that only 19% had nuts more than three times per week. Oil use was recorded by two studies conducted in the Mediterranean region, i.e., Spain and Italy [23,32] and both observed that people with T2DM tended to use olive oil instead of butter. Fortes et al. [23] observed that nearly 90% of participants reported that they have never used butter, or using less than once per week, while nearly 95% reported the exclusive use of olive oil. Munoz-Pareja et al. [32] observed that 90% had less than one serving of butter, margarine, or cream per day, but 88.3% reported using olive oil for cooking.

## 4. Discussion

This systematic review examined, for the first time, the KAB in relation to dietary fat of people with T2DM and their healthcare professionals. Overall, people with T2DM seemed to have poor knowledge regarding dietary fat, while their attitudes and behaviors towards dietary fat were highly heterogenous. These results are concerning as they imply potential limitations for people to self-manage their condition, which is critical in achieving optimal long-term health outcomes and reducing the risk of developing diabetes-related complications [43].

Our findings indicated that people with T2DM in the included studies were unable to accurately identify foods that were high in fat and unable to recognize the link between dietary fat intake and blood biomarkers, indicating a disconnect between their diet and health outcomes. Previous meta-analyses found that intervention programs that enhanced participants’ knowledge related to T2DM led to better glycemic control and quality-of-life outcomes [44,45], as well as saved cost when compared with the routine primary care for T2DM [46]. As such, given that diabetes posed a substantial financial burden to people with diabetes and health systems [47], diabetes education delivered to people with T2DM is a cost-effective way to improve their well-being in the long-term [48]. In particular, the intake of dietary fat subtypes had great implications for patients’ lipid profiles and risk of cardiovascular diseases and is one strategy that can be utilized to improve outcomes [49]. Educational materials regarding dietary interventions for diabetes should strengthen these areas to enhance patients’ understanding regarding the effects of dietary fat (both amount and types) on their T2DM management outcomes.

The attitudes of study participants towards dietary fat were highly heterogenous, with observations made for favoring both low and high fat intake. Moreover, we observed that both the general public and dietitians expressed confusion over whether individuals with T2DM should follow a low- or high-fat diet. This could be a result of drastic changes in recommendations related to dietary fat, which switched from the low-fat paradigm advocated in 1990s to a focus on sources of fat in recent dietary guidelines [50]. While it is expected that participants in older studies would perceive fat as a nutrient to be avoided [33], it is alarming to find that participants in more recent studies share the same belief [21,31]. In line with this, clinical practice guidelines also showed variations in the recommendation towards dietary fat intake related to T2DM treatment [51]. Since healthcare professionals are perceived by the public as a trustworthy source of information [52], their lack of confidence would fuel the confusion among the general public and highlights the importance of incorporating a multidisciplinary team to holistically manage T2DM. In particular, dietitians should be considered routinely as part of the diabetes management team to act as a source of tailored and individualized dietary information, instead of relying on clinicians to provide such information, which may not be part of their expertise and/or where there are limitations on time during consultations [53].

The intake of saturated fat among participants in most of the included studies were higher than 10% of total energy as recently recommended by the American Diabetes Association [49]. This is worrying as high-saturated-fat intake was associated with adverse cardiovascular outcomes in people with T2DM [54]. Furthermore, high saturated fat intake is linked to increased inflammation in individuals with diabetes; this was demonstrated in a study showing that a meal rich in fat produces a further increase in TNF-alpha levels associated with endothelial dysfunction and oxidative stress, which are the pathophysiological substrate of diabetes complications [55]. Another recommendation related to fat intake in people with T2DM concerns the adoption of a Mediterranean eating pattern, which is rich in plant-based mono- and polyunsaturated fat [2]. However, adherence related to this recommendation is hard to assess in this systematic review as food group intakes were measured only in a few of the included studies, which were conducted in Mediterranean regions. In these studies, while olive oil was commonly used in cooking, nuts were only consumed by a small proportion of individuals with T2DM, implying limited plant-based fat intake. It would be beneficial for future studies to collect data on food group intake, so that study results can be compared with guidelines for a more objective assessment. It is also important to note that while participants were aware that saturated fat has an adverse impact on their health, much less about the participants’ KAB towards unsaturated fat was documented in the published studies. Given the beneficial effects of unsaturated fat in T2DM management and prevention [3,4,49], more research in this regard is warranted as it could serve as a positive reinforcement for the encouraging consumption of unsaturated fat [56]. This would also convey a message to the general public about the beneficial aspect of dietary fat consumption instead of solely focusing on the adverse effect of saturated fat, thus reducing the confusion around dietary fat and the avoidance of it altogether and providing a beneficial dietary alternative [50].

One strength of this systematic review includes the assessment of KAB towards dietary fat in people with T2DM, which is the first review of its kind and thus provides novel insights regarding the current state of KAB related to diabetes. On the other hand, there are several limitations to acknowledge. Firstly, the research methods used to assess knowledge and attitudes were heterogenous, thereby leading to results that may not be comparable between studies. Second, only one study involved healthcare professionals, and therefore, this was removed from the study outcomes; however, it highlights a key gap in the literature. Third, a meta-analysis was not possible due to variations in the methods of recording nutrient intake and statistical analysis among the included studies. Fourth, some of the earlier studies included did not collect data on participants’ attitudes towards fat subtypes. As a result, findings related to fat subtypes could only be synthesized from a subgroup of studies. Finally, the KAB related to dietary fat was influenced by culture and food availability, yet given the limited number of studies, we were unable to synthesize culture-specific findings.

## 5. Conclusions

In this systematic review, we found that people with T2DM had poor knowledge and highly varied attitudes towards dietary fat. Saturated fat intake, in general, was higher than recommended. Future studies are needed to assess the KABs towards dietary fat subtypes and related food groups in those with T2DM and healthcare professionals alike, given the vital role dietary management plays in overall diabetes management. This is essential to guide the development of dietary management guidelines for diabetes management.

## Figures and Tables

**Table 1:** PICOS question.

Population	Adults with Type 2 Diabetes Mellitus
Intervention	High-Healthy-Fat Diet
Comparator	Not Applicable
Outcome	Knowledge, Attitudes, and Behaviors

**Table 2:** Search terms used in this systematic review.

Concept	Alternatives (OR)
Type 2 diabetes	diabet*, t2d, t2dm, niddm, ‘non-insulin dependent diabetes mellitus’, TIIDM
AND	
Dietary fats	Fat*, saturated, mufa, pufa, sfa, ‘Mediterranean diet’, nut*, avocado*, fish, ‘olive oil*’, seed*, monounsaturated, polyunsaturated, linoleic, linolenic, DHA, ALA, EPA, oil*, keto*, ‘oleic acid’, omega, ‘long chain omega’, lcn*
AND	
Knowledge, attitude, behavior/practice	Know*, attitude*, behav*, belief*, perception*, practice*, confidence*, provision*, prescription*, experience*, affect*, value*, abilit*, feel*

**Table 3:** Quality assessment of included studies.

Study	Relevance Questions				Validity Questions										Outcome
	1	2	3	4	1	2	3	4	5	6	7	8	9	10	
Breen et al. 2015 [18]	NA	Y	Y	Y	Y	Y	NA	Y	N	Y	Y	Y	Y	Y	Positive
Devi et al. 2021 [19]	Y	Y	Y	Y	Y	Y	NA	NA	N	Y	U	N	N	Y	Neutral
Di Onofrio et al. 2018 [20]	Y	Y	Y	Y	Y	U	N	N	N	Y	Y	Y	Y	Y	Neutral
Ewers et al. 2021 [21]	Y	Y	Y	Y	Y	Y	Y	N	Y	Y	Y	Y	Y	Y	Positive
Fitzgerald et al. 2008 [22]	NA	Y	Y	NA	Y	Y	Y	N	N	Y	Y	Y	Y	Y	Positive
Fortes et al. 2021 [23]	Y	Y	Y	Y	Y	Y	NA	Y	N	Y	Y	Y	Y	Y	Positive
Gaurthier-Chelle et al. 2004 [24]	NA	Y	Y	NA	Y	U	Y	N	Y	N	Y	Y	Y	Y	Neutral
Gebeyehu et al. 2022 [25]	Y	Y	Y	NA	Y	Y	NA	Y	NA	Y	Y	Y	Y	Y	Positive
Hendrychova et al. 2013 [26]	NA	Y	Y	NA	Y	Y	NA	Y	N	Y	Y	Y	Y	Y	Positive
Hendrychova et al. 2015 [27]	NA	Y	Y	NA	Y	Y	Y	N	N	Y	U	Y	Y	Y	Neutral
Ismael et al. 2021 [28]	Y	Y	Y	Y	Y	Y	NA	Y	U	Y	Y	Y	Y	Y	Positive
Lindstrom et al. 2006 [29]	Y	Y	Y	Y	Y	Y	NA	Y	NA	Y	Y	Y	Y	Y	Positive
Melnik et al. 2006 [30]	NA	Y	Y	NA	Y	Y	NA	Y	N	Y	Y	Y	Y	Y	Positive
Mphwanthe et al. 2021 [31]	Y	Y	Y	Y	Y	Y	NA	N	N	Y	U	Y	N	Y	Neutral
Muñoz-Pareja et al. 2012 [32]	NA	Y	Y	NA	Y	Y	NA	N	Y	Y	Y	Y	Y	Y	Positive
Parker et al. 1995 [33]	NA	Y	Y	NA	Y	Y	Y	Y	N	Y	Y	Y	Y	Y	Positive
Quandt et al. 2009 [34]	NA	Y	Y	NA	Y	Y	NA	Y	N	Y	Y	Y	Y	Y	Positive
Taylor et al. 2014 [35]	NA	Y	Y	NA	Y	U	NA	N	NA	Y.	Y	Y	Y	Y	Neutral
Thewjitcharoen et al. 2018 [36]	NA	Y	Y	NA	Y	Y	NA	Y	NA	Y.	Y	Y	Y	Y	Positive
Vasconcelos et al. 2021 [37]	Y	Y	Y	Y	Y	U	NA	Y	U	U	Y	N	Y	Y	Neutral
Webster et al. 2019 [38]	NA	Y	Y	NA	Y	N	NA	Y	N	Y	Y	Y	Y	N	Neutral
Wong et al. 2021 [39]	Y	Y	Y	Y	Y	N	NA	N	N	Y	Y	Y	Y	Y	Neutral
Xue et al. 2019 [40]	Y	Y	Y	Y	Y	Y	NA	Y	U	Y	U	N	Y	Y	Neutral

Quality assessment was conducted using the Academy of Nutrition and Dietetics Evidence Analysis Library Quality Criteria Checklist. Y, yes (green). N, no (red). U, unclear (yellow). NA, not applicable. A “positive” was given if answers to validity questions 2, 3, 6, 7, and an additional question are “yes”, or else a “neutral” was given.

## Supplementary Materials

The following supporting information can be downloaded at: https://www.mdpi.com/article/10.3390/nu16142185/s1, Table S1: PRISMA 2020 Checklist. Reference [57] is cited in the supplementary materials.

## Abbreviations

Type 2 diabetes mellitus (T2DM); Type 1 diabetes mellitus (T1DM); Monounsaturated fatty acids (MUFA); Polyunsaturated fatty acids (PUFA); Knowledge, Attitudes and Behaviors (KAB); PRISMA (Preferred Reporting Items for Systematic Reviews and Meta-analysis).
